# Active control of all-fibre graphene devices with electrical gating

**DOI:** 10.1038/ncomms7851

**Published:** 2015-04-21

**Authors:** Eun Jung Lee, Sun Young Choi, Hwanseong Jeong, Nam Hun Park, Woongbin Yim, Mi Hye Kim, Jae-Ku Park, Suyeon Son, Sukang Bae, Sang Jin Kim, Kwanil Lee, Yeong Hwan Ahn, Kwang Jun Ahn, Byung Hee Hong, Ji-Yong Park, Fabian Rotermund, Dong-Il Yeom

**Affiliations:** 1 Department of Physics and Department of Energy Systems Research, Ajou University, 206 Worldcup-ro, Yeongtong-gu, Suwon 443-749, Republic of Korea; 2 Soft Innovative Materials Research Center, Korea Institute of Science and Technology (KIST), 92 Chudong-ro, Bongdong-eup, Jeonbuk 565-905, Republic of Korea; 3 Department of Chemistry, Seoul National University, 1 Gwanak-ro, Gwanak-gu, Seoul 151-747, Republic of Korea; 4 Nano Photonics Research Center, KIST, 5, Hwarang-ro 14-gil, Seongbuk-gu, Seoul 136-791, Republic of Korea

## Abstract

Active manipulation of light in optical fibres has been extensively studied with great interest because of its compatibility with diverse fibre-optic systems. While graphene exhibits a strong electro-optic effect originating from its gapless Dirac-fermionic band structure, electric control of all-fibre graphene devices remains still highly challenging. Here we report electrically manipulable in-line graphene devices by integrating graphene-based field effect transistors on a side-polished fibre. Ion liquid used in the present work critically acts both as an efficient gating medium with wide electrochemical windows and transparent over-cladding facilitating light–matter interaction. Combined study of unique features in gate-variable electrical transport and optical transition at monolayer and randomly stacked multilayer graphene reveals that the device exhibits significant optical transmission change (>90%) with high efficiency-loss figure of merit. This subsequently modifies nonlinear saturable absorption characteristics of the device, enabling electrically tunable fibre laser at various operational regimes. The proposed device will open promising way for actively controlled optoelectronic and nonlinear photonic devices in all-fibre platform with greatly enhanced graphene–light interaction.

## Abstract (web summary)

Active control of light in optical fibres is of great interest, to this end, electric control of all-fibre graphene devices is desirable but highly challenging. Here, Lee *et al*. demonstrate electric control of the optical properties of a graphene sheet deposited on a side-polished fibre mediated by an ion liquid.

## Graphical abstract

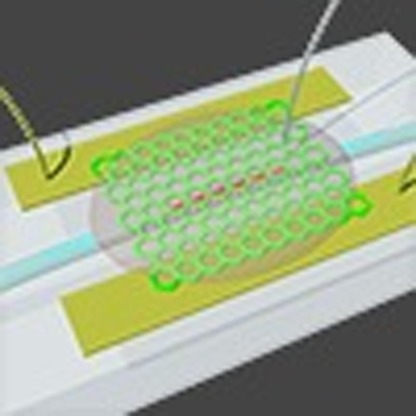

The extraordinary electrical and optical properties of graphene have attracted strong interest in the fields of optoelectronics, plasmonics and photonics applications^1^,^2^,^3^. In particular, its uniform linear absorption and huge nonlinearity over a broad spectral range enable the advent of novel photonic devices such as broadband polarizers^4^, wavelength-independent nonlinear wavelength converters^5^,^6^, broadband nonlinear saturable absorbers (SAs)^7^,^8^ and optical limiters^9^. One of the fascinating features of graphene is its optical transition properties substantially modified with electrical signals^10^,^11^,^12^, which allows electrically controllable photonic devices with great flexibility. For example, optical modulators have been demonstrated by electrically tuning the Fermi level of a graphene layer integrated with silicon waveguides^13^,^14^ or photonic crystal nanocavity^15^ reporting a maximum modulation depth up to 10 dB at resonant wavelength, but at the expense of excessive coupling loss. All-fibre photonic devices that indirectly interact with graphene by evanescent-field coupling are expected to potentially exhibit enhanced graphene–light interaction, a high optical damage threshold, and low insertion loss with fibre-optic communications and fibre laser systems, but previous researches on the topic are restricted to passive devices with limited performance^4^,^8^,^16^. Despite previous theoretical suggestion^2^, gate-controlled graphene devices in optical fibres have yet to be realized mainly because significant optical loss is unavoidable by the metal electrode placed near the graphene/optical waveguide in conventional gating geometry.

In this work, we demonstrate electrically controllable all-fibre graphene devices for the first time. The graphene-based field effect transistor (FET) was built on a side-polished fibre (SPF) with an ion-liquid dielectric. Here the ion-liquid dielectric plays a key role as an extremely efficient electrical gating medium^17^. The ion-liquid dielectric, as a transparent over-cladding, also greatly increases the graphene–light interaction with negligible absorption loss (<0.03 dB). The electrical transport characteristics and the related optical transition properties of the device are simultaneously investigated for high-quality large-area monolayer and stacked multilayer graphene. The fabricated all-fibre graphene device exhibits a markedly large optical transmission change (>90%) with low insertion loss at the applied gate voltage of <3 V. Furthermore, the device was successfully integrated into a fibre laser system as an electrically tunable in-line nonlinear SA, where the laser operation can be tuned from continuous wave, through Q-switched to passively mode-locked regime with electrical signals.

## Results

### Design and fabrication of the devices

**Figure 1:**
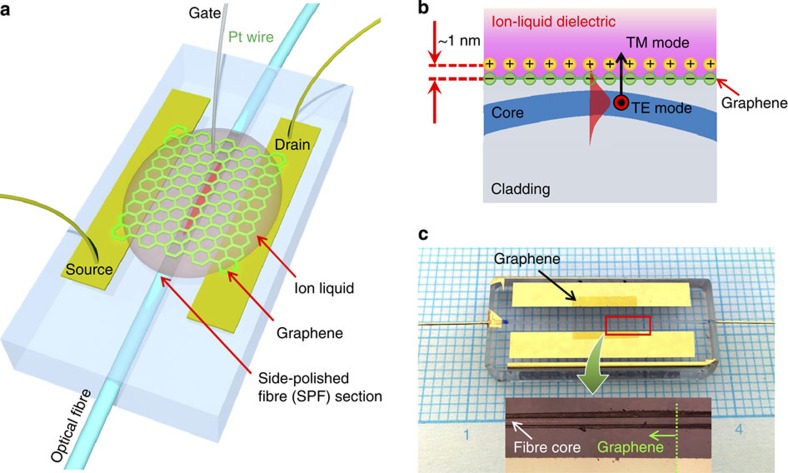
Schematic view and images of gate-controlled all-fibre graphene devices. (**a**) Schematic diagram of gate-variable all-fibre graphene device. The SPF was fabricated using a standard single-mode optical fibre (SMF-28e) where two metal electrodes were deposited with 50-nm thickness at both sides of the side-polished region. After transferring the graphene layer, ion liquid was applied to the graphene. Two electrodes and a Pt wire were used as source, drain and gate, respectively. The electrical transport property as a function of applied gate voltages was monitored through the measurement of *I*_D_. Optical power in the fibre was measured using a laser diode at 1,550 nm, an in-line polarization controller and an optical power metre. (**b**) Schematic of side view of the all-fibre graphene devices. The field tail of the guided mode interacts with the graphene. Ion liquid acts as the gate medium as well as a transparent cladding that enhances the field strength at the graphene surface. The applied gate voltage makes the ion form an EDL between the ion liquid and graphene with a thickness of about 1 nm, which efficiently modifies the Fermi level in graphene. (**c**) Optical image of the fabricated device without ion liquid. The graphene sheet (7 × 5 mm^2^) covers the region of interaction with the fibre (∼5 mm). Inset: microscope image of side-polished surface where red light was launched from fibre end to visualize the core at polished surface.

All-fibre graphene devices were fabricated on the SPF as shown schematically in Fig. 1a. An optical fibre buried into the quartz block was side-polished and two metal electrodes were subsequently deposited on the quartz block, serving as source and drain (see Methods section). A high-quality large-area monolayer graphene grown using the chemical vapour deposition (CVD) method^18^ and its stacked multilayers were transferred onto the side-polished region of the fibre (see Methods) for evanescent-field interaction with the guided mode in the optical fibre. A droplet of ion liquid (CAS-No: 174899-82-2, Merck Millipore, Germany) was then applied to the device, covering the graphene layers, and a Pt wire was employed as a gate electrode. When the gate voltage is applied, the ions form an electric double layer (EDL) at the liquid/graphene interface with effective capacitance thickness around 1 nm over entire area of graphene as shown in Fig. 1b. The extremely high capacitance of the electric double layer leads to a significant change of the Fermi level of graphene, which substantially modifies the optical transmission of the light in the optical fibre at low operation voltage. The evanescent wave selectively couples with the graphene layer for different input polarizations because of the geometrical asymmetry of the device. We experimentally confirmed that the transverse electric (TE) mode whose polarization is parallel to the graphene surface dominantly interacts with the graphene layer (see Supplementary Fig. 1). Figure 1c shows a photographic image of the fabricated device. The interaction length of the side-polished region is estimated as 5 mm, while a 7 × 5-mm^2^ graphene layer covers the side-polished section. A magnified image of the interaction region is shown in the inset of Fig. 1c, where the fibre core on the polished surface is displayed by launching red light into the fibre. The graphene sheet uniformly covers the interaction section of the SPF and part of the metal electrodes.

### Gate-variable properties of all-fibre graphene devices

**Figure 2:**
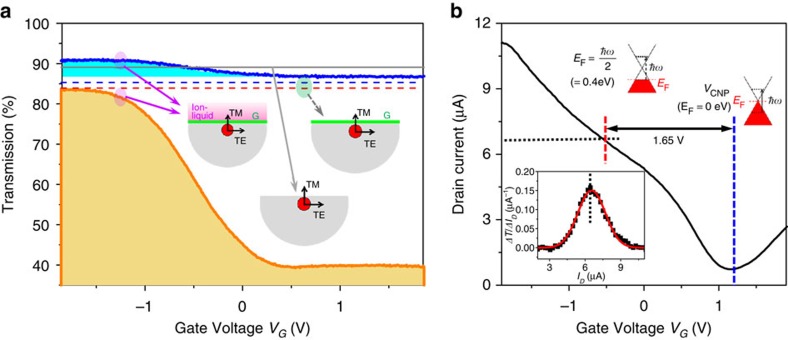
Gate-variable properties of the all-fibre graphene device using a monolayer graphene. (**a**) Optical transition properties of the device. Initial fibre-to-fibre insertion loss of the SPF was 0.5 dB (grey solid line). The insertion loss slightly increased by 0.2 and 0.27 dB for TM and TE mode, respectively, after transferring the monolayer graphene (blue and red dash lines). Blue and orange solid lines show gate-variable optical transmission for TM and TE mode, respectively, after applying the ion liquid. (**b**) Electrical transport properties of the device. The on–off *I*_D_ current ratio was 16.7 for the applied *V*_G_ range of ±1.8 V. The estimated *V*_CNP_ was 1.16 V (blue vertical dash line) where the Fermi level of graphene is close to zero. Inset: normalized change of optical transmission (Δ*T*/Δ*I*_D_) as a function of drain current. The experimental result (black solid square) is displayed with fitted line (red solid line). Maximum of transmission change occurs at *I*_D_ of 6.55 μA (corresponding gate voltage is −0.49 V (red vertical dash line)) where the Fermi energy level shift is half the incident photon energy.

Figure 2 represents the gate-voltage-dependent properties of the device fabricated using a monolayer graphene. The SPF initially exhibits fibre-to-fibre connection loss of 0.5 dB (grey solid line) as shown in Fig. 2a, which is mainly due to the scattering loss from the surface roughness of the polished section. After transferring the monolayer graphene on the SPF, we observed that the insertion loss increases by 0.2 dB with polarization dependent loss of 0.07 dB as indicated by the horizontal dashed lines (blue: transverse magnetic (TM) mode, red: TE mode). When the ion liquid with refractive index of about 1.423 (ref. 19) is applied on the graphene, strong graphene–light interaction occurs owing to enhanced evanescent-field strength at the graphene layer (Supplementary Fig. 2). At zero gate bias voltage (*V*_G_), the TE mode suffers strong absorption of 3.47 dB (orange solid line at zero *V*_G_), whereas transmission of the TM mode increases by 0.09 dB (blue solid line at zero *V*_G_). This is because the ion-liquid over-cladding alleviates the scattering loss caused by the graphene and the polished surface. More importantly, optical transmission of the device was actively tuned with an applied gate voltage. As shown in Fig. 2a, the TM transmission changes from 87.1 to 90.8% for *V*_G_ ranging from 0.7 V to −1.8 V. The TE mode shows a more drastic transmission change from 39.2 to 83.4% for the same *V*_G_ range. This indicates that 44.2% of total incident light recoverably interacts with the monolayer graphene for the TE mode. It is worth noting that the total insertion loss (<1 dB) in our device is much smaller than that of previously reported silicon waveguide-based device (25 dB)^14^ or even in-line graphene polarizer (∼15 dB)^4^. In particular, use of a high-quality uniform graphene layer covered by transparent ion-liquid cladding results in a substantially lower scattering loss (<0.5 dB) during interaction with the graphene layer than others (typically 4–5 dB)^4^,^14^.

A shift of Fermi level in graphene is responsible for this optical transmission change. Limited density of state of electrons confined in a two-dimensional graphene leads to a significant change of the Fermi level near the Dirac point with the applied gate bias, resulting in strong electro-optic absorption^10^,^11^,^12^, which is not observed in normal bulk materials. The variation of electron carrier density in graphene was studied by measuring electrical transport properties of the device. The ambipolar transfer characteristics of the monolayer graphene FET on the SPF is displayed in Fig. 2b at a drain voltage (*V*_D_) of 2 mV. The drain current (*I*_D_) varies with *V*_G_ where the on–off current ratio was measured as 16.7 within the applied *V*_G_ of ±2 V. The voltage at charge neutral point (*V*_CNP_) appears to be 1.16 V (blue vertical dash line), which indicates that the graphene on the quartz substrate is initially p-doped. The inset of Fig. 2b shows the transmission change (Δ*T*) of the TE mode with respect to the drain current variation (Δ*I*_D_). The experimental results (black solid square) and the fitted data (red solid line) clearly reveal that the derivative has a maximum at *I*_D_=6.55 μA (black dotted line), which corresponds to an applied gate voltage of −0.49 V (red vertical dash line). The maximum change in optical transmission will occur when the Fermi level approaches half the photon energy 
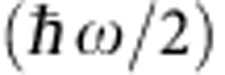
. Thus, the gate voltage difference 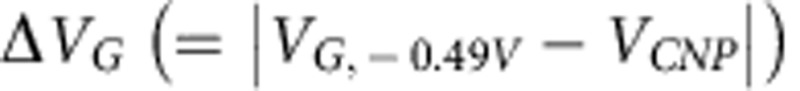 of 1.65 V directly corresponds to the Fermi level shift of 0.40 eV, which is the half photon energy of the light source used (1,550 nm) in the present experiment. These combined optical and electrical measurements can provide an alternative way to estimate the important parameters such as gate coupling efficiency^20^ and quantum capacitance^21^ of the graphene device without using sophisticated electrical measurement systems. It is worth noting that the refractive index and the attenuation coefficient simultaneously vary by applying gate voltages on graphene. We numerically calculated the change of both the refractive index and the attenuation coefficient of graphene as a function of Fermi energy (see Supplementary Note 1). Although the refractive index can considerably change with the Fermi-level shift, we do not expect any significant contribution to the optical transmission change because graphene layer is too thin to modify the condition to couple the radiation mode with the refractive index change in the current scheme.

**Figure 3:**
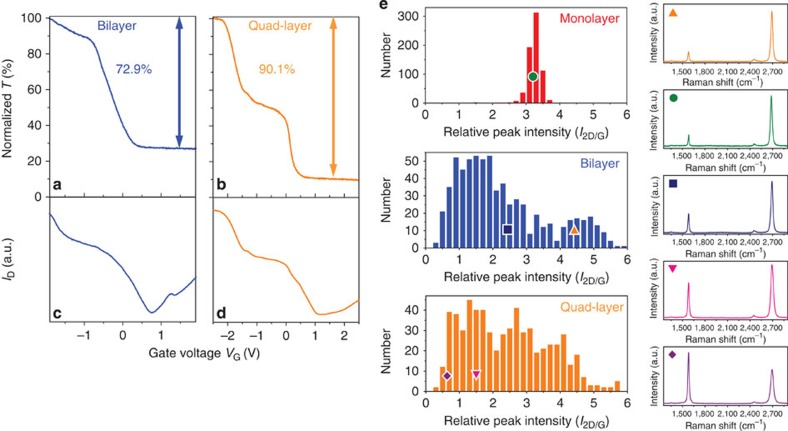
Device performance and statistical analysis of Raman spectrum in multilayer graphene. Gate-controlled optical properties of all-fibre devices with (**a**) bi- and (**b**) quad-layer graphenes. Stronger graphene–light interaction leads to enhanced non-resonant optical transmission change of 72.9% (bilayer) and 90.1% (quad-layer). Corresponding electrical transport properties of devices with (**c**) bi- and (**d**) quad-layer graphenes. (**e**) Statistical analysis of Raman spectroscopic measurements. Histograms show the relative peak intensity of the Raman signal between 2D-peaks and G-peaks (*I*_2D/G_). The Raman spectra at several sampling points are shown in the figures on the right side. The monolayer graphene has an almost uniform ratio of 2.9–3.6. The results of multilayer graphene exhibit broad distribution, which indicates that there is significant interlayer coupling in our stacked multilayer graphene.

The simple structure of the proposed device readily allows it to be extended to multilayer graphene-based systems, providing an opportunity to enhance device efficiency and to explore the electro-absorption features of multilayer graphene. Large-area bi- and quad-layer graphenes prepared with a multiple stacking method^18^ are transferred onto the SPF. Figure 3a,b compares the optical properties of the device with bilayer and quad-layer graphenes as a function of applied gate voltage, respectively, where the optical transmission of the TE mode is normalized with respect to its maximum. In the measurement, applied gate voltage ranges were different depending on the number of graphene layers to prevent the occurrence of a chemical reaction. The device with bilayer graphene shows a stronger interaction than that with a monolayer graphene, resulting in an optical transmission change of 72.9%. Moreover, in the case of quad-layer graphene, the linear absorption markedly varies up to 90.1% for the operation voltage of <3 V. Corresponding electrical transport properties are shown in Fig. 3c (bilayer) and Fig. 3d (quad-layer). Interestingly, it is clearly observed that the carrier transport and the optical transmission indicate step-like responses to gate voltage. To study this behaviour, Raman spectroscopic measurement was carried out over the area (50 × 50 μm^2^) of the graphene samples (see Supplementary Fig. 3). Figure 3e summarizes the results, where statistical analysis of relative Raman signal intensity between the G-peaks and 2D-peaks (*I*_2D/G_) obtained from Raman images are depicted for mono-, bi- and quad-layer graphenes, respectively, from top to bottom (left). Shown together are the corresponding Raman spectra at several sampling points (right). The monolayer graphene has a nearly constant *I*_2D/G_ of around 2.9–3.6 over the most area (>95%). For multilayer graphene samples, the histograms extracted from the Raman images indicate that only small portion of films (6.3% for bilayer and 10.1% for quad-layer) demonstrates the similar *I*_2D/G_ to that of the monolayer graphene. In these areas, electrical properties of each graphene layer are expected to be preserved owing to the weak layer-to-layer interaction^18^. However, a substantial portion of the distribution has *I*_2D/G_ less than that of monolayer graphene, which indicates that there is a significant interlayer coupling even in our randomly stacked multilayers similar to Bernal-stacked graphene^22^. In these regions, additional conduction channels with interband scattering can open at high carrier densities, which allow further increase of the drain current at the higher gate voltages instead of current saturation observed in a monolayer graphene^23^. This enables second step in the optical response with optical transmission changes of >90% as shown in Fig. 3b. Different initial doping states for different layers^24^, or a screening effect^25^ in multiple-stacking graphene layers may also partially contribute to this step-like response.

### Electrically tunable fibre laser operation

**Figure 4:**
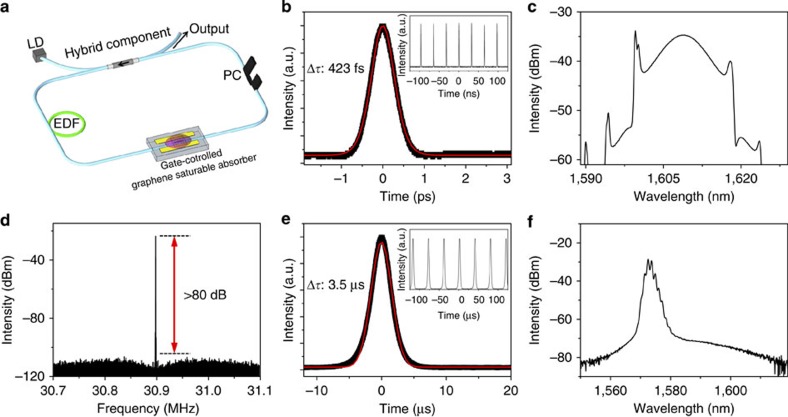
Fibre laser operation using the all-fibre graphene device. Gate-variable properties of fibre laser operation. (**a**) Fibre laser configuration including fabricated all-fibre device with bilayer graphene. (**b**–**d**) Characteristics of passively mode-locked fibre laser at an applied *V*_G_ of −1.05 V; (**b**) Measured pulse duration of 423 fs at a repetition rate of 30.9 MHz (inset). (**c**) Laser output spectrum with a spectral bandwidth of 8 nm at 3 dB. (**d**) Measured radio frequency spectrum of the laser output (**e**) and (**f**) Q-switched characteristics of fibre laser at an applied *V*_G_ of −0.18 V; (**e**) Measured output pulse duration of 3.5 μs at a repetition rate of 25.4 kHz (inset) and (**f**) its optical spectrum.

The linear optical transmission change with gate voltage can also lead to the modification of the optical nonlinear properties of graphene. In particular, nonlinear saturable absorption characteristics of graphene such as saturation fluence and nonlinear modulation depth can be continuously tuned with the gate voltage, which can overcome the discrete nature of nonlinear absorption in stacked graphene sheet. We measured the nonlinear optical transmission of our device, which varies with the applied gate voltage (Supplementary Fig. 4). With an advantage of fibre compatibility, our device could be easily integrated into an all-fibre laser system. To experimentally confirm the feasibility of the device to apply as an electrically controllable nonlinear in-line SA, we built all-fibre Er-doped laser by incorporating our SA with bilayer graphene as shown in Fig. 4a (see Methods section). The fibre laser shows a self-starting of passive mode-locking operation at the applied *V*_G_ of −1.05 V where the measured pulse duration was 423 fs at a repetition rate of 30.9 MHz as shown in Fig. 4b and its inset. The spectral bandwidth of the laser output was measured to be 8.0 nm at the central wavelength of 1,609 nm (Fig. 4c) where the average output power was 3.1 mW. The background noise level was <80 dB from the signal of fundamental repetition rate of the laser output as shown in Fig. 4d, which indicates stable mode-locking operation. The laser operation was stably maintained during the experiment of few hours. On increasing *V*_G_, the linear optical transmission decreases, while both modulation depth and saturation fluence of the SA increase. This significantly modifies the Q-switching instability condition^26^,^27^, changing the fibre laser operation to Q-switching. Figure 4e,f shows measured Q-switched pulse duration (3.5 μs) and optical spectrum, respectively, at the applied *V*_G_ of −0.18 V. The repetition rate of the laser was measured as 25.4 kHz (inset of Fig. 4e). For an applied voltage larger than 0.14 V or less than −1.65 V, the fibre laser turns to continuous wave operation. The performance of the device is well sustained for the repeated experiments over the period of several months without replenishing the ion liquid because ion liquids typically exhibit negligible vapour pressure at room temperature^28^. Our results well support the transition behaviour between the several regimes of pulsed lasers^26^,^27^, where modulation depth of SA is one of the most crucial parameters for a switchable operation. We expect that such switchable fibre laser operation can be considered as a suitable solution for developing versatile pulse laser seed sources, which can potentially find a number of applications both in Q-switching and mode-locking regimes (for example, distance measurement, photo-acoustic imaging, remote sensing, time-resolved spectroscopy or optical frequency metrology). Furthermore, it might be interesting to study the noise properties including timing jitter in a passively mode-locked fibre laser with controlled carrier dynamics properties of a graphene for various gate voltages.

## Discussion

In conclusion, we have demonstrated, for the first time to our knowledge, electrically tunable all-fibre graphene devices with extremely low insertion loss by fabricating the graphene-based FET on the SPF. Here the ion liquid critically acts as a highly efficient gating medium as well as a transparent over-cladding that greatly increases graphene–light interaction without significant optical loss. By combined study of electric transport and optical transmission properties of the device with a monolayer graphene and its stacked multilayers, we realize all-fibre graphene devices capable of controlled large optical transmission change (>90%) at a low operation voltage (<3 V). Furthermore, electrically manipulated nonlinear absorption properties of our devices enable it to apply as a tunable in-line graphene SA, which can electrically control the fibre laser operation at different regimes as follows: continuous wave, Q-switching and passive mode-locking. Although its gating speed is currently limited by the ionic mobility in the ion liquid^29^, the proposed graphene device will immediately find enormous applications particularly in electrically controlled nonlinear optics^5^,^6^,^7^,^8^,^9^, broadband photodetection^30^,^31^ and control of graphene carrier dynamics^32^ in an all-fibre platform with greatly enhanced interaction with graphene. In addition, the suggested concept can be further extended to an optical fibre taper that potentially provides more tight confinement of light, resulting in reduced device size with higher operation speed of the device. The proposed scheme can be also applied to the plastic optical waveguides combined with an ionic gel, paving a novel way for actively controlled, flexible photonic devices.

## Methods

### Device fabrication

A conventional single-mode fibre (SMF-28e) was embedded into the quartz block with a radius curvature of 250 mm and side-polished until the insertion loss increased to −60 dB in the presence of the index oil with a refractive index of 1.458. The insertion loss of the SPF was 0.5 dB without index oil. Cr/Au electrodes with a thickness of 50 nm (Cr: 5 nm and Au: 45 nm) were deposited using an e-beam evaporator and a metal mask. Graphene monolayer films were grown on 25-μm-thick copper foils via low-pressure thermal CVD . The copper foil was cut into 10 × 10-cm^2^ size, cleaned using nitric acid for few seconds, rinsed off with deionized water to remove any unwanted particles on the both surfaces of the foil and rolled into the fused silica chamber of the CVD furnace. Then the copper foil was heated at 1,000 °C under the flow rate of 20 sccm of H_2_ for 30 min, and CH_4_ gas was allowed to flow into the chamber with 50 sccm for 30 min. After completing the thermal process, the furnace was cooled down to room temperature. The poly(methyl methacrylate) (PMMA) was spin-coated on the as-grown graphene with a thickness of 300 nm as a supporting layer, which is then cut to desired sizes after removing the graphene layer grown on the other side of the copper foil by a reactive-ion etching method. The copper foils were etched using ammonium persulphate aqueous solution. For the randomly stacked few-layer graphene films, the PMMA/graphene films were directly transferred on to the monolayer graphene-grown copper foil without additional PMMA film and the etching process was repeated. The PMMA-coated graphene films were then transferred onto the SPF and the PMMA support layer was dissolved in an acetone bath for an hour and rinsed with isopropyl alcohol.

### Fibre laser experiment

A ring cavity fibre laser was built as shown in Fig. 4a. Highly Er-doped fibre with a length of 40 cm was used as a gain medium and the gain fibre was optically pumped by a 976-nm laser diode. A hybrid component acting as a wavelength division multiplexing coupler, an optical isolator and an output coupler with 10% output coupling ratio were inserted into the laser cavity. The polarization state in the laser cavity was adjusted by a polarization controller. Finally, our all-fibre graphene device was inserted in the fibre laser cavity as a gate-variable SA where the laser output properties such as optical spectrum, pulse duration, repetition rate and output power were monitored while varying the gate voltage.

## Author contributions

D.-I.Y. conceived and designed the experiment. S.Y.C., M.H.K., S.S., S.B., S.J.K., F.R. and B.H.H. prepared the graphene samples. E.J.L., S.Y.C., H.J. and N.H.P. fabricated the graphene devices. E.J.L., H.J., N.H.P., W.Y., J.-Y.P. and D.-I.Y. tested the performance of the devices. J.-K.P. and Y.H.A. measured the Raman signal. K.L., Y.H.A., K.J.A., J.-Y.P., F.R. and D.-I.Y. prepared the manuscript.

## Additional information

How to cite this article: Lee E. J. *et al*. Active control of all-fibre graphene devices with electrical gating. *Nat. Commun.* 6:6851 doi: 10.1038/ncomms7851 (2015).

## Supplementary Material

Supplementary Information Supplementary Figures 1-4, Supplementary Note 1 and Supplementary Reference

## References

[b1] Bonaccorso F., Sun Z., Hasan T. & Ferrari A. C. Graphene photonics and optoelectronics. Nat. Photonics 4, 611–622 (2010).

[b2] Bao Q. & Loh K. P. Graphene photonics, plasmonics, and broadband optoelectronic devices. ACS Nano 6, 3677–3694 (2012). 22512399 10.1021/nn300989g

[b3] Avouris P. Graphene photonics, plasmonics, and optoelectronics. IEEE J. Sel. Top. Quantum Electron. 20, 6000112 (2014).

[b4] Bao Q. . Broadband graphene polarizer. Nat. Photonics 5, 411–415 (2011).

[b5] Hendry E., Hale P. J., Moger J., Savchenko A. K. & Mikhailov S. A. Coherent nonlinear optical response of graphene. Phys. Rev. Lett. 105, 097401 (2010). 20868195 10.1103/PhysRevLett.105.097401

[b6] Xu B., Martinez A. & Yamashita S. Mechanically exfoliated graphene for four wave-mixing based wavelength conversion. IEEE Photonics Technol. Lett. 24, 1792–1794 (2012).

[b7] Bao Q. . Atomic-layer graphene as a saturable absorber for ultrafast pulsed lasers. Adv. Funct. Mater. 19, 3077–3083 (2009).

[b8] Choi S. Y. . All-fiber dissipative soliton laser with 10.2 nJ pulse energy using an evanescent field interaction with graphene saturable absorber. Laser Phys. Lett. 11, 015101 (2014).

[b9] Lim G. K. . Giant broadband nonlinear optical absorption response in dispersed graphene single sheets. Nat. Photonics 5, 554–560 (2011).

[b10] Wang F. . Gate-Variable Optical Transitions in Graphene. Science 320, 206–209 (2008). 18339901 10.1126/science.1152793

[b11] Li Z. Q. . Dirac charge dynamics in graphene by infrared spectroscopy. Nat. Phys. 4, 532–535 (2008).

[b12] Zhang Y. . Direct observation of a widely tunable bandgap in bilayer graphene. Nature 459, 820–823 (2009). 19516337 10.1038/nature08105

[b13] Liu M. . A graphene-based broadband optical modulator. Nature 474, 64–67 (2011). 21552277 10.1038/nature10067

[b14] Liu M., Yin X. & Zhang X. Double-layer graphene optical modulator. Nano Lett. 12, 1482–1485 (2012). 22332750 10.1021/nl204202k

[b15] Gan X. . High-contrast electrooptic modulation of a photonic crystal nanocavity by electrical gating of graphene. Nano Lett. 13, 691–696 (2013). 23327445 10.1021/nl304357u

[b16] Song Y. W., Jang S. Y., Han W. S. & Bae M. K. Graphene mode-lockers for fiber lasers functioned with evanescent field interaction. Appl. Phys. Lett. 96, 051122 (2010).

[b17] Fujimoto T. . Electric-double-layer field-effect transistors with ionic liquids. Phys. Chem. Chem. Phys. 15, 8983–9006 (2013). 23665738 10.1039/c3cp50755f

[b18] Bae S. . Roll-to-roll production of 30-inch graphene films for transparent electrodes. Nat. Nanotechnol. 5, 574–578 (2010). 20562870 10.1038/nnano.2010.132

[b19] Buffeteau T. . Imidazolium-based ionic liquids: quantitative aspects in the far-infrared region. J. Phys. Chem. B 114, 7587–7592 (2010). 20469889 10.1021/jp102087m

[b20] Heller I. . Comparing the weak and strong gate-coupling regimes for nantotube and graphene transtiors. Phys. Status Solidi RRL 3, 190–192 (2009).

[b21] Xia J., Chen F., Li J. & Tao N. Measurement of the quantum capacitance of graphene. Nat. Nanotechnol. 4, 505–509 (2009). 19662012 10.1038/nnano.2009.177

[b22] Ferrari A. C. . Raman spectrum of graphene and graphene layers. Phys. Rev. Lett. 97, 187401 (2006). 17155573 10.1103/PhysRevLett.97.187401

[b23] Ye J. . Accessing the transport properties of graphene and its multilayers at high carrier density. Proc. Natl Acad. Sci. USA 108, 13002–13006 (2011). 21828007 10.1073/pnas.1018388108 PMC3156196

[b24] Lee S. K. . Stretchable graphene transistors with printed dielectrics and gate electrodes. Nano Lett. 11, 4642–4646 (2011). 21973013 10.1021/nl202134z

[b25] Sul Y. & Appenzeller J. Screening and interlayer coupling in multilayer graphene field-effect transistors. Nano Lett. 9, 2973–2977 (2009). 19639984 10.1021/nl901396g

[b26] Keller U. . Semiconductor saturable absorber mirrors (SESAM's) for femtosecond to nanosecond pulse generation in solid-state lasers. IEEE J. Sel. Top. Quantum Electron. 2, 435–453 (1996).

[b27] Hönninger C., Paschotta R., Morier-Genoud F., Moser M. & Keller U. Q-switching stability limits of continuous-wave passive mode locking. J. Opt. Soc. Am. B 16, 46–56 (1999).

[b28] Zhang S., Sun N., He X., Lu X. & Zhang X. Physical properties of ionic liquids: database and evaluation. J. Phys. Chem. Ref. Data 35, 1475–1517 (2006).

[b29] Ozel T. . Polymer electrolyte gating of carbon nanotube network transistors. Nano Lett. 5, 905–911 (2005). 15884892 10.1021/nl0503781

[b30] Xia F., Mueller T., Lin Y., Valdes-Garcia A. & Avouris P. Ultrafast graphene photodetector. Nat. Nanotechnol. 4, 839–843 (2009). 19893532 10.1038/nnano.2009.292

[b31] Gan X. . Chip-integrated ultrafast graphene photodetector with high responsivity. Nat. Photonics 7, 883–887 (2013).

[b32] Breusing M. . Ultrafast nonequilibrium carrier dynamics in a single graphene layer. Phys. Rev. B 83, 153410 (2011).

