# Supplementary material for: Active control of all-fibre graphene devices with electrical gating
Source: Nat Commun. 2015 Apr 21;6:6851. doi: 10.1038/ncomms7851 (PMC4410643; doi:10.1038/ncomms7851)
Supplement: Supplementary Information — Supplementary Figures 1-4, Supplementary Note 1 and Supplementary Reference [file ncomms7851-s1.pdf]

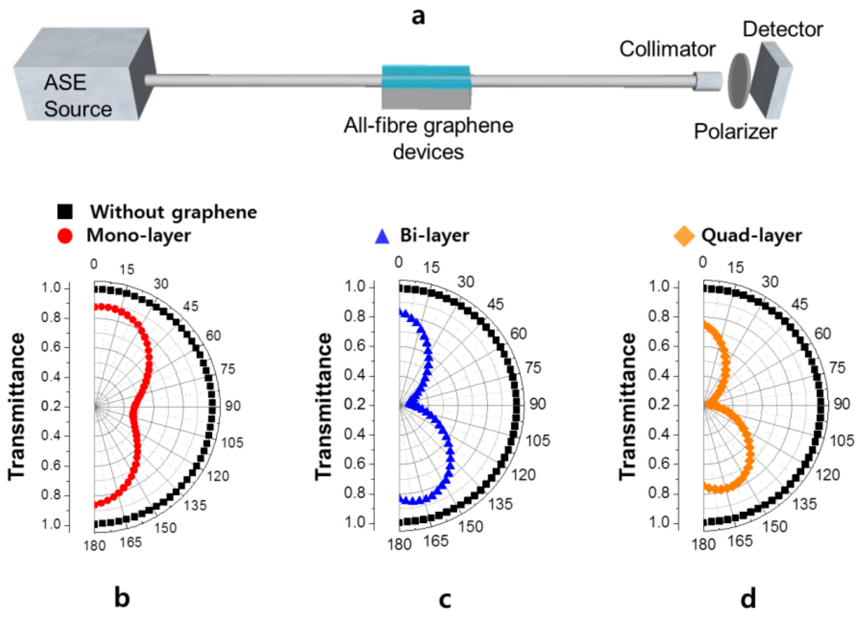

Supplementary Figure 1: Experimental measurement of polarization-dependent absorption properties in all-fibre graphene devices. **a.** Schematic of experimental set-up including an amplified spontaneous emission (ASE) source around 1550 nm, an in-line collimator, a linear polarizer, and a photo-detector. **b.- d.** Angular plot of optical transmission of the devices. **b.** The device with a mono-layer graphene shows the maximum transmission (~ 88%) at 7.6° with minimum transmission (~ 46%) at almost normal (97.1°) to that of maximum where TE-polarization direction corresponds to 90° degree **c.** Same measurement with bi-layer graphene shows the maximum transmission (~ 86%) at 167° with minimum transmission (~ 27%) at 77.5°. **d.** The angles at maximum (~ 78%) and minimum transmission (~ 24%) were measured to be 163.7° and 74.5°, respectively, for quad-layer sample. In all samples, the angle at minimum transmission is randomly distributed around 90 ± 15° and the angle at maximum is nearly normal at the accuracy within ± 1°. A shift of the angle from the polarization direction of TE- and TM-modes is expected to be originated from the induced birefringence by the SPF buried into the quartz block with a curvature and the magnetic block to hold the optical fibres. The devices without graphene show nearly angle-independent transmission (black square).

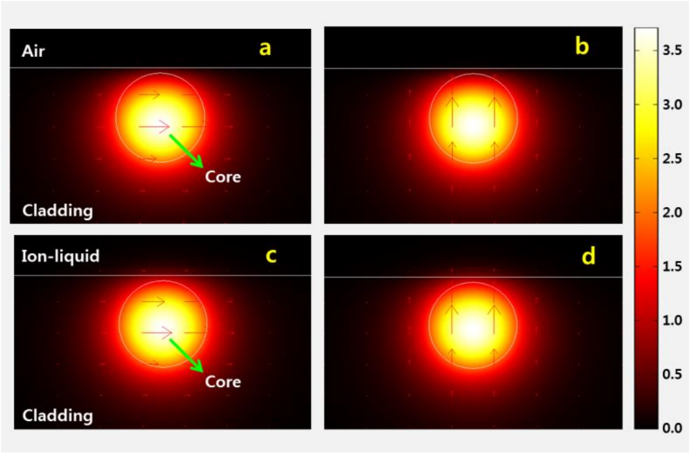

Supplementary Figure 2: Numerical calculation of field enhancement on the SPF surface by ion-liquid. The electric field distribution and polarization direction of the fundamental guided mode in the SPF were numerically calculated using a commercial software (COMSOL Multiphysics®) where the optical fibre, core-cladding index difference and minimum distance between core-boundary and polished surface were set as 8.3 μm, 0.36%, and 0.5 μm, respectively. The electric field distribution and polarization direction of **a.** TE- and **b**. TM-mode without ion-liquid. The relative intensity ratio at polished surface with respect to maximum intensity ($I_{surface}/I_{max}$) was 0.36% and 0.17% for TE- and TM-mode, respectively. **c.** TE- and **d.** TM-mode with ion-liquid. The calculated $I_{surface}/I_{max}$ was 4.05% and 4.00% for TE- and TM-mode respectively.

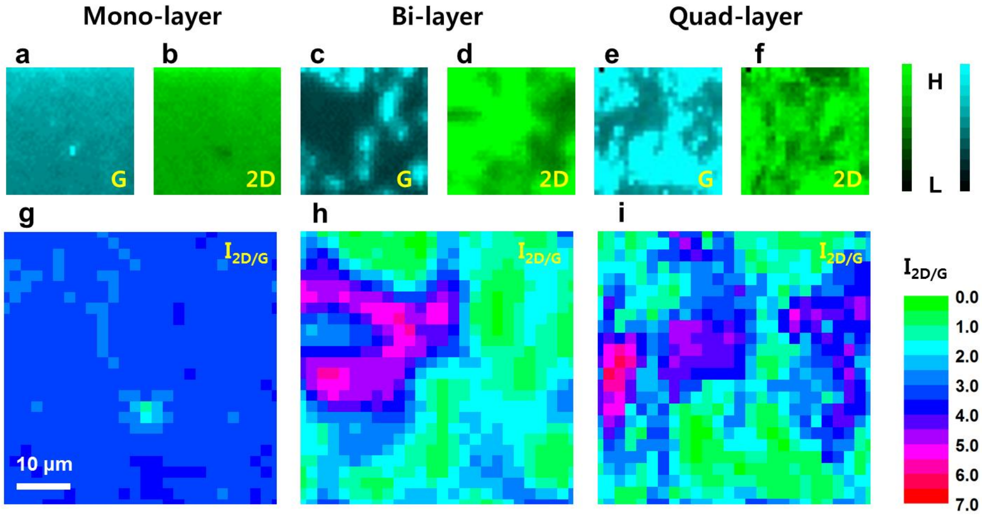

Supplementary Figure 3: Raman measurement and images in randomly stacked graphene layers. Spatial distribution of relative Raman intensity between 2D-peak and G-peak ($I_{2D/G}$) recorded by a commercial Raman microscopy (inVia Raman microscope, Renishaw plc). A light with central wavelength of 514 nm was focused on the graphene samples using an objective lens (Numerical Aperture = 0.5, 20X). Raman spectra at 729 (= 27×27) different points have been taken over the area of 50×50 μm^2 of the individual graphene samples with a focused spot size of ~ 1 μm^2. **a.-f.** Raman intensity image around G peak and 2D peak of the sample (**a.** and **b.** mono-layer graphene, **c.** and **d.** bi-layer graphene, **e.** and **f.** quad-layer graphene) **g.-i.** Calculated $I_{2D/G}$ distribution of **g.** mono-layer graphene, **h.** bi-layer graphene and **i.** quad-layer graphene samples extracted from each measurement. While the monolayer graphene exhibits almost uniform distribution of $I_{2D/G}$ 2.9 ~ 3.6 over the most area, the $I_{2D/G}$ in multilayer graphene samples show broad distribution ranging from 0.4 to 6. In particular, they hold smaller $I_{2D/G}$ than that of mono-layer graphene over substantial area (73.4% and 64.3% for bi- and quad-layer graphenes, respectively) of the sample.

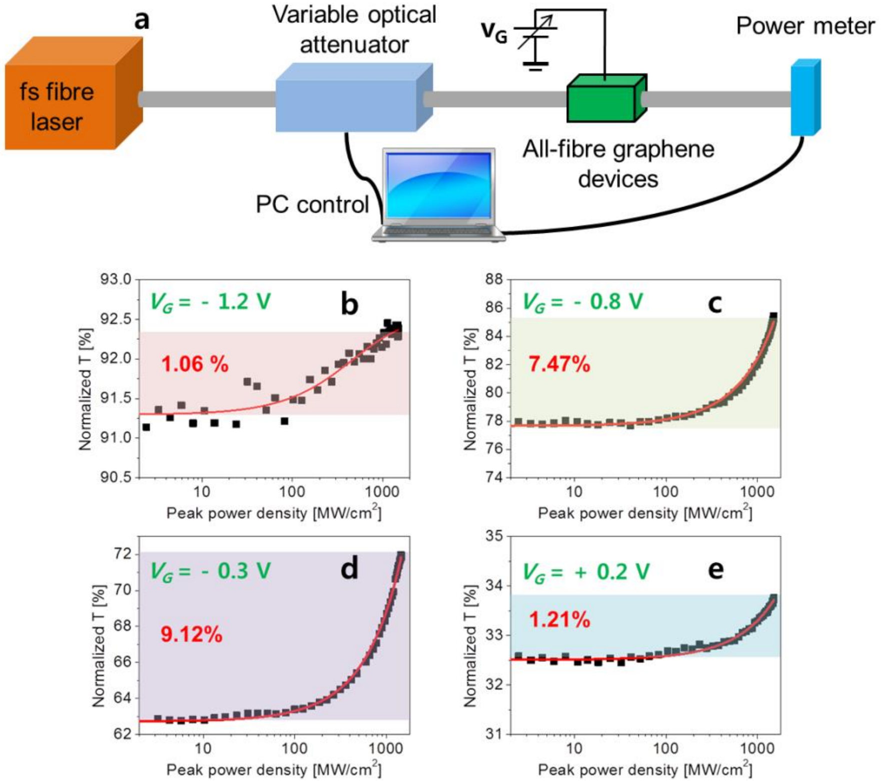

Supplementary Figure 4: Gate-variable nonlinear transmission properties of TE mode in the all-fibre graphene device using a bi-layer graphene. a. Schematic of nonlinear measurement setup using a lab-built mode-locked fibre laser, a polarization controller, a variable optical attenuator, and an optical power meter. **b.-e.** Normalised nonlinear transmission curves at the applied gate voltages of **b.** − 1.2 V, **c.** − 0.8, **d.** − 0.3 and **e.** + 0.2 V. The nonlinear fitting (red solid line) of experimental results (black solid square) shows the modulation depth of 1.06 % with a saturation fluence of 256.6 MW/cm^2 at the V_G of − 1.2 V. There is more significant nonlinear optical transmission change for V_G of – 0.8 V (7.47 %) and − 0.3 V (9.12%) though the absorption could not be fully saturated due to the limit of currently available input power of the source. In case of V_G at + 0.2 V, only 1.21% of the nonlinear optical transmission change was observed for a given maximum input power because of the limited input power and increased saturation fluence of the graphene SA.

Supplementary Note. 1: Dielectric constant of graphene depending on Femi-energy

We calculated the dielectric constant of graphene $\varepsilon_g(\omega)$ from[1]

$$\varepsilon_g(\omega) = \varepsilon_\infty + \frac{i\sigma(\omega)}{\varepsilon_0 \omega d}, \qquad (1)$$

where ε_∞ (= 2.5) is the background dielectric constant of graphene, ε_0 vacuum permittivity, d (= 0.335 nm) the graphene thickness. The frequency ω and Femi-energy E_F dependent optical conductivity $\sigma(\omega)$ is obtained with

$$\sigma(\omega) = \frac{2e_0^2 k_B T}{\pi\hbar^2}\frac{i}{\omega + i\Gamma}\log(2\cosh(\frac{E_F}{2k_B T})) + \frac{e_0^2}{4\hbar^2}\left\{H\left(\frac{\omega}{2}\right) + \frac{4i\omega}{\pi}\int_0^\omega d\epsilon \frac{H(\omega) - H\left(\frac{\epsilon}{2}\right)}{\omega^2 - 4\epsilon^2}\right\}, \qquad (2)$$

$$H(\epsilon) = \frac{\sinh(\hbar\epsilon/k_B T)}{\cosh(E_F/k_B T) + \cosh(\hbar\epsilon/k_B T)}, \qquad (3)$$

where ε_0 denotes the elementary charge, k_B the Boltzmann constant, T temperature, $\hbar$ Plank constant, and Γ the decay rate of electron plasma. By using this model we obtained the refractive index (n) and the attenuation coefficient (κ) as a function of Fermi energy at the wavelength of 1.55 μm. For example, as the Fermi energy increases from 0 to 0.4 eV, the complex refractive index of graphene varies from 3.2165 + 2.622i to 3.3689 + 2.2777i.

Supplementary Reference

1. Falkovsky, L. A. Optical properties of graphene. *J. Phys. Conf. Ser.* **129**, 012004, (2008).
